# Who Worries More?: Generational Differences in Climate Anxiety in South Korea

**DOI:** 10.1093/geroni/igaf122.2285

**Published:** 2025-12-31

**Authors:** Giyeon Kim, Takashi Yamashita, Daniel Jimenez

**Affiliations:** Chung-Ang University, Seoul, Republic of Korea; University of Maryland, Baltimore County, Baltimore, Maryland, United States; University of Miami Miller School of Medicine, Miami, Florida, United States

## Abstract

Climate change poses a growing threat to public health, disproportionately impacting older adults who are more vulnerable to extreme weather events such as heatwaves and floods. The present study aims to examine generational differences in climate anxiety among South Korean adults using nationally representative data from the 2024 Social Survey. We analyzed climate change prevalence and associated factors across three age groups: young adults (aged 20-39), middle-aged adults (aged 40-59), and older adults (aged 60 and older). Results indicate a significant increase in climate anxiety from 2022 to 2024, with 53.2% of respondents expressing anxiety. Among them, 41.1% reported moderate anxiety, while 12.1% experienced severe anxiety. Climate anxiety levels showed generational differences: Climate anxiety levels were higher among middle-aged adults (57.5%) and younger adults (52.15%) than older adults (50.3%). Additionally, across all three age groups, those who are younger, women, and living in urban areas tended to have higher levels of climate anxiety. This suggests possible generational differences in risk perception, adaptation, or psychological resilience. As climate-related disasters become more frequent, these findings underscore the need for targeted public health interventions. Policies should prioritize psychological resilience-building and developing age-sensitive risk communication strategies. Future research should explore coping mechanisms, long-term mental health impacts, and policy implications for different generations in the context of climate challenges.

